# Supplementary material for: Metformin reverses early cortical network dysfunction and behavior changes in Huntington’s disease
Source: eLife. 2018 Sep 4;7:e38744. doi: 10.7554/eLife.38744 (PMC6156080; doi:10.7554/eLife.38744)
Supplement: Transparent reporting form [file elife-38744-transrepform.docx]

***eLife’s* transparent reporting form**

We encourage authors to provide detailed information *within their submission* to facilitate the interpretation and replication of experiments. Authors can upload supporting documentation to indicate the use of appropriate reporting guidelines for health-related research (see [EQUATOR Network](http://www.equator-network.org/%20)), life science research (see the [BioSharing Information Resource](https://biosharing.org/)), or the [ARRIVE guidelines](http://www.plosbiology.org/article/info:doi/10.1371/journal.pbio.1000412) for reporting work involving animal research. Where applicable, authors should refer to any relevant reporting standards documents in this form.

If you have any questions, please consult our Journal Policies and/or contact us: [editorial@elifesciences.org](mailto:editorial@elifesciences.org).

**Sample-size estimation**

- You should state whether an appropriate sample size was computed when the study was being designed
- You should state the statistical method of sample size computation and any required assumptions
- If no explicit power analysis was used, you should describe how you decided what sample (replicate) size (number) to use

Please outline where this information can be found within the submission (e.g., sections or figure legends), or explain why this information doesn’t apply to your submission:

We conducted a power analysis for determining the minimum sample size for *in vivo* calcium imaging, resulting in a minimum of 5 mice for each experimental group. This sample size is sufficient to determine differences in frequency of calcium transients of 30% at an alpha level of 0.05 with a power (1-ß) of 0.8 using a two-tailed fixed effects analysis of variance (ANOVA) (G*Power3 analysis based on an effect size of 2.2 which was derived from a previous study using the same methodology (Busche et al.2008).In the present manuscript, we used 8 WT mice ( n=1204 cells);6 Hdh150 mice (n=933 cells); 9 metformin-treated WT mice (n=1915 cells) and 6 metformin-treated Hdh150 mice (n=1585 cells).

In the open field behavioral tests, we first performed a priory power analysis with a small test cohort. As we observed a strong effect, the number of at least 6 mice per group in the Open field was chosen.

### For Western blot analysis we used the customary number of biological replicates ranging between 3 and 6 (e.g. Al-Gharaibeh et al., 2017).

**Replicates**

- You should report how often each experiment was performed
- You should include a definition of biological versus technical replication
- The data obtained should be provided and sufficient information should be provided to indicate the number of independent biological and/or technical replicates
- If you encountered any outliers, you should describe how these were handled
- Criteria for exclusion/inclusion of data should be clearly stated
- High-throughput sequence data should be uploaded before submission, with a private link for reviewers provided (these are available from both GEO and ArrayExpress)

Please outline where this information can be found within the submission (e.g., sections or figure legends), or explain why this information doesn’t apply to your submission:

The table 1 of the methods section states the number of biological replicates. For *in vivo* calcium imaging analysis, the number of neurons analyzed was provided in addition.

We provide the following information in the method section:

Numbers of biological replicates are provided as number of mice, worms or cells used in the different experiments. For immunofluorescence experiments, a technical replicate of three per animal was employed.

No technical replicates were obtained in calcium imaging data, filter retardation assays, FRAP-based assays and Western blots.

No other data points were excluded.

**Statistical reporting**

- Statistical analysis methods should be described and justified
- Raw data should be presented in figures whenever informative to do so (typically when N per group is less than 10)
- For each experiment, you should identify the statistical tests used, exact values of N, definitions of center, methods of multiple test correction, and dispersion and precision measures (e.g., mean, median, SD, SEM, confidence intervals; and, for the major substantive results, a measure of effect size (e.g., Pearson's r, Cohen's d)
- Report exact p-values wherever possible alongside the summary statistics and 95% confidence intervals. These should be reported for all key questions and not only when the p-value is less than 0.05.

Please outline where this information can be found within the submission (e.g., sections or figure legends), or explain why this information doesn’t apply to your submission:

Table 1 contains all details concerning statistical tests used (name of the test, p-values, F values and degree of freedom).

We tested all datasets for normality using the one-sample Kolmogorov-Smirnov test. In case that the null hypothesis of a normal distribution could not be rejected (for p>0.05), we employed a parametric test, if H0 could be rejected (for p<0.05) we used non-parametric tests: Mann-Whitney U test for non-parametric data and t test for parametric data

Raw data: Examples of OGB-1 AM staining are shown in Figure 1c, Figure 1 – figure supplement 1b and Figure 5 - figure supplement 1a. Representative western blots are shown in Figure 4b, 4e and 4g. A representative example of filter retardation assay is shown in Figure 4a.Representative examples of trajectories in mouse behavioral tests are shown in Figure 3a and in Figure 5h.

(For large datasets, or papers with a very large number of statistical tests, you may upload a single table file with tests, Ns, etc., with reference to sections in the manuscript.)

**Group allocation**

- Indicate how samples were allocated into experimental groups (in the case of clinical studies, please specify allocation to treatment method); if randomization was used, please also state if restricted randomization was applied
- Indicate if masking was used during group allocation, data collection and/or data analysis

Please outline where this information can be found within the submission (e.g., sections or figure legends), or explain why this information doesn’t apply to your submission:

In behavioral experiments, the experimenter was blinded with regard to the genotype and treatment.

**Additional data files (“source data”)**

- We encourage you to upload relevant additional data files, such as numerical data that are represented as a graph in a figure, or as a summary table
- Where provided, these should be in the most useful format, and they can be uploaded as “Source data” files linked to a main figure or table
- Include model definition files including the full list of parameters used
- Include code used for data analysis (e.g., R, MatLab)
- Avoid stating that data files are “available upon request”

Please indicate the figures or tables for which source data files have been provided:

We added a file containing the code used for the analysis of the calcium imaging data.

We included a table with numerical data depicted for each figure as source data.
